# Effect of Acupressure at Sanyinjiao Point 6 on pain in primary dysmenorrhea among medical students at a private institute in Karachi

**DOI:** 10.12669/pjms.41.4.10818

**Published:** 2025-04

**Authors:** Samira Amjad, Qamer Aziz, Ruqaya Nangrejo, Mubushra Samina

**Affiliations:** 1 Samira Amjad, MCPS, M Phil Department of Physiology, Karachi Institute of Medical Sciences, Malir Cantonment, Karachi, Pakistan; 2 Qamer Aziz, PhD Department of Physiology, Baqai Medical University, Karachi Pakistan; 3 Ruqaya Nangrejo, PhD Department of Physiology, Baqai Medical University, Karachi Pakistan; 4 Mubushra Samina, FCPS Department of Gynaecology, Karachi Institute of Medical Sciences, Malir Cantonment, Karachi, Pakistan

**Keywords:** Acupressure, Primary dysmenorrhea, Sanyinjiao point- 6, Visual Analog Scale

## Abstract

**Objective:**

This study aimed to determine the difference in pain intensity before and after acupressure at Sanyinjiao Point 6 (SP6) in medical students with Primary Dysmenorrhea (PD).

**Methods:**

The research design was quasi-experimental adopting a single group comparing pre and post-intervention data. The study was conducted in a private medical institute in Karachi, Pakistan, from September 2022 to April 2023. To select participants, a modified menstrual symptom questionnaire was used, and to measure the pain, the Visual Analogue Scale (VAS) was used during the four months of the study. A convenient sample of 50 healthy medical students were included who suffered from PD and were not using analgesics. After taking informed written consent, in the first month, VAS scoring was done without intervention on the first three days of the menstrual cycle (MC). In the subsequent three MCs, acupressure was self-applied (after being taught by a qualified acupressurist) three times a day at acupoint SP6 for two to five minutes on both legs one by one during the first three days of each intervention MC and VAS pain scoring was done.

**Results:**

A statistically significant reduction in pain intensity (p<0.01) was observed in post-acupressure VAS scores during the first three days of each intervention menstrual cycle.

**Conclusion:**

Acupressure at the SP6 point has been shown to effectively reduce pain intensity in medical students with primary dysmenorrhea. As a result, this alternative therapy can be recommended as a non-pharmacological option for pain management.

## INTRODUCTION

The term “Dysmenorrhea” is derived from the Greek words “dys”, “meno” and “rrhea” meaning difficult/unusual/painful, month, and flow respectively.^1^ Pain due to Menstrual Cycles (MC) is known as dysmenorrhea, without any pelvic pathology it is called Primary Dysmenorrhea (PD), and when associated with pelvic pathology it is termed as Secondary.^1^ In gynaecological clinics, PD is found to be the most common presenting complaint.

According to Hippocrates, pain during menstruation is due to cervical canal obstruction causing stagnation of blood. The fact that nulliparous women have greater pain as compared to multiparous women is supportive of this theory.^2^ PD is usually found in ovulatory cycles only which generally do not begin immediately after menarche.^3^ Prostaglandins (PG) cause dysmenorrhea is the well-accepted theory regarding its pathophysiology, PGE2 and PGF2 are found to be released during sloughing of the endometrium causing ischemia, decreased blood flow, abnormal uterine contractions, and inflammation.^4^

Dysmenorrhea is associated with many factors such as dietary intake; coffee^3^, sweet snacks, carbonated soft drinks, and fast foods have shown a positive association.^5^ Conversely, healthy fats may reduce cramps^1^,^6^, as omega-3 has shown to lower PG production by incorporating into membrane phospholipids.^6^ Other risk factors include a positive family history, a higher body mass index, early menarche, and longer, heavier MCs.^7^ A meta-analysis including countries of all socioeconomic statuses, concluded a high prevalence irrespective of the economic condition of the country.^1^ Cultural differences can lead to differences in reporting of pain due to differences in pain perception and threshold.^8^

The prevalence of Dysmenorrhea is underestimated and hard to establish as most women do not seek medical help. Other reasons include the absence of a standard for describing the severity of pain and the variability in the definition of the term dysmenorrhea.^6^ According to various studies, the prevalence in different parts of the world is as follows: Romania 78.4%^2^, Kuwait 85.6%^3^, Spain 85.2%^9^, Sweden 89%^10^, and Pakistan 78.6%.^7^

In a Spanish study, a lack of concentration in class or work was reported by 51.3%, while 62.8% reported a loss of academic work.^9^ A study in Pakistan showed that 33% of PD sufferers required medical consultation and that only 65% used analgesics, revealing 35% as not using analgesics.^7^ Another study in Sweden reported that 63% of women found no relief with analgesics for dysmenorrheic pain.^10^ Acupressure is a Traditional Chinese Medicine (TCM) technique where physical pressure is applied on particular points on the body (known as acupoints) using either a thumb, finger, or device.^11^ According to TCM, there are energy (Qi) channels in the body called meridians which are of two opposing types Yin and Yang.^12^

According to previous studies, acupressure increases endogenous analgesics via autonomic nervous stimulation.^12^ Acupressure stimulates the skin causing the release of endorphins that relax the body and block the pain-sensing receptors in the brain.^13^ This study aimed to provide a safe, low-cost, and non-invasive method for women to reduce the pain of PD. This study analyzed the effects of acupressure, on pain during PD, which to the best of our knowledge, has not yet been conducted in Pakistan. A large number of women are averse to taking analgesics during menstruation due to various myths and beliefs, hence acupressure may prove to be an effective therapy for pain relief. The objective of this study was to observe the difference in pain during PD, before and after applying acupressure at SP6 using the Visual Analogue Score (VAS).

## METHODS

This study is quantitative research, using a quasi-experimental design that includes a single group with a pre-intervention and post-intervention approach. Data was collected between September of 2022 and April 2023 from students of a private medical college in Karachi. The majority of participants were hostelers providing ease for teaching acupressure and follow-up of acupressure application during their MCs. Data collection was spread over six months.

### Ethical Approval:

It was obtained by the Ethical Committee of Baqai Medical University. The reference number is BMU-EC/04-2022, dated Sept. 16, 2022. Fifty medical students were included in the study.

### Inclusion criteria:

Healthy females between ages 16 and 25 years old with normal, regular menstruation, suffering PD every month, and not using analgesics. All World Health Organization Body Mass Index categories were included.

### Exclusion criteria:

Those having any pelvic disease, history of pelvic surgery, depression or mental illness, a recent stressful incident, diabetes mellitus, hypertension, or any other co-morbid conditions, injury in the ankle area, or any neuropathies. The students were selected based on a modified menstrual symptom questionnaire distributed to 250 medical students from all academic years.

### Sample size:

The sample size was calculated by a statistician using a Confidence Interval of 95% and a Power of 80, a total sample size of 20 before, and 20 after intervention participants was calculated. To strengthen the study, instead of 20, we used 50 participants from whom both pre and post-intervention data was obtained.

### Materials:

i) A modified menstrual questionnaire was used to select students suffering from primary dysmenorrhea every month and not using any pharmacological method for pain relief. ii) A data proforma consisting of the Visual Analog Score (VAS) to be recorded over four MCs. The VAS was selected as it is one of the frequently used self-reported measures for pain evaluation, has proven very feasible for research in the clinical domains, and is not cumbersome for the participant and researcher.^14^ The proforma also recorded the duration and frequency of acupressure applied in each MC.

### Procedure:

**Fig.1:**
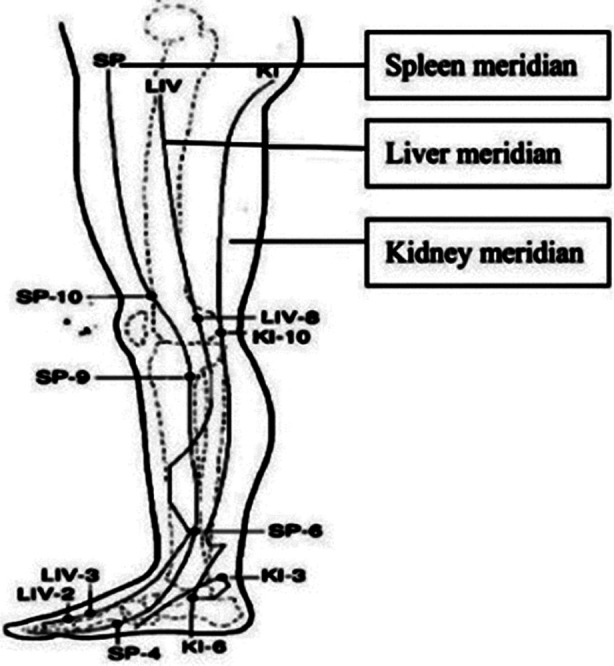
Sanyinjiao Point 6, intersection of three yin meridians.^16^ (San Yin Jiao means “intersection of three yin meridians”).^16^ (Reproduced with kind permission from Wong et al, 2010).

The participants were informed in detail about the study and a written consent was taken. They were asked to note down the pain intensity on the VAS on the first three days of their menstrual cycle. In the first month, there was no intervention. The participants were taught how to perform acupressure at SP6 under the guidance of a qualified acupressurist. This point is located four fingers above the medial malleolus between the muscle and tibia bone^15^ as shown in Fig.1. Acupressurist-approved videos were also shared with the participants for their convenience. They were requested to apply constant pressure at the SP6 acupoint with their finger or thumb three times a day on both legs for two to five minutes (according to their comfort) during dysmenorrhea. This was done in the subsequent three menstrual cycles. The participants noted the intensity of pain on the VAS proforma after applying acupressure. They were also requested not to use any other alternative method for pain relief during the study months.

**Table-I:** Baseline characteristics of the studied participants (n=50).

Parameters	Mean	SD
Age (years)	20.92	1.56
Weight (kg)	54.6	10.8
Height (m)	1.615	.063
BMI (kg/m^2^)	20.91	3.60
Age of Menarche (years)	13.28	1.52
Number of bleeding days (days)	5.30	1.38

## RESULTS

Data was submitted by all 50 participants and was stored and analyzed using SPSS version 23.0. The mean and Standard Deviation (SD) of the baseline characteristics were reported. The Mean and SD for the VAS scores for pain on Day one, Day two, and Day three in the study’s first (pre-acupressure), second, third, and fourth (post-acupressure) months were reported. Paired sample t-test was used to compare pre-acupressure and post-acupressure VAS mean scores. Pearson correlation test was used to correlate BMI and other baseline characteristics with VAS. P-values less than 0.05 were considered statistically significant.

**Table-II:** Comparison of the Pre- and Post-Acupressure VAS Scores Across the First, Second, And Third Months.

Day	Pre-acu VAS	Post-acu VASMonth-1	p-value	Post-acu VAS Month-2	p-value	Post-acu VASMonth-3	p-value
	Mean±SD	Mean±SD		Mean±SD		Mean±SD	
Day-1	7.14±1.38	5.76±1.57	<0.01*	5.74±1.93	<0.01*	5.42±2.03	<0.01*
Day-II	5.36±1.72	4.32±1.72	<0.01*	3.881.76	<0.01*	3.98±1.94	<0.01*
Day-III	2.80±1.88	2.08±1.53	<0.01*	1.821.56±	<0.01*	1.76±1.57	<0.01*

* p<0.05 was considered statistically significant using the Paired Sample t-test Pre-acu: Pre-acupressure, Post-acu: Post-acupressure.

**Table-III:** Correlation Analysis of Baseline Parameters with Pre-Acupressure VAS.

Parameters		Age (years)	BMI (kg/m2)	Age of Menarche (years)	Number of bleeding days
Pre Acupressure day-1 VAS	r-value	0.005	0.056	0.000	-0.012
	p-value	0.971	0.699	0.998	0.936
Pre Acupressure day-2 VAS	r-value	-0.163	0.089	0.031	0.014
	p-value	0.257	0.540	0.832	0.925
Pre Acupressure day-3 VAS	r-value	-0.241	-0.019	-0.058	0.016
	p-value	0.092	0.894	0.688	0.914

*p<0.05 was considered statistically significant for correlation.

## DISCUSSION

The significance of this research is hinged on the necessity of guidance for women regarding alternative management of dysmenorrhea as it affects various aspects of their daily lives. It has been shown previously that ethnicity is related to pain perception^8^, hence it is important to note that this type of study, to the best of our knowledge, is the first of its kind in Pakistan i.e. studying the Pakistani population. To prevent, screen, and treat PD is the responsibility of midwives, and as reported by Midwifery Regulation, the substitution of complementary medicine can reduce treatment costs.^17^

The current study provides insight into the field of acupressure at SP6 in PD for pain relief. PD has been widely studied ranging from its risk factors to a wide variety of management options. Since PD is prevalent and women are willing to accept alternative self-practiced methods, we need to promote low-cost, safe, easy, and effective methods for pain relief. Systematic reviews have shown that acupressure is *as effective as pharmacological therapy* for relief of dysmenorrhea.^18^ Acupressure at SP6 is reported to be one of the most favoured alternative methods for pain relief in dysmenorrhea.^19^ Pakistani studies regarding acupressure for PD were not found, however, studies other than acupressure, such as the relationship between junk food and menstrual abnormalities^5^, the impact of dysmenorrhea on academics^7^, and other such studies have been conducted. Since a single group was used to compare the pre and post-acupressure VAS scores, it makes the study more authentic as the different demographic characteristics and also pain threshold were the *same before and after* the intervention.

The VAS scores of this study showed statistically significant differences in the pre-acupressure and post-acupressure with p<0.01. Pain relief was found to be statistically significant in all three days (p<0.01) during the three intervention MCs. The VAS pain scores decreased significantly irrespective of the duration of acupressure applied i.e. varying between two to five minutes. Equally effective were the different frequencies of pressure application i.e. twice or thrice a day. While comparing with other studies, different durations, frequencies, and types of acupressure were found. Some studies used constant pressure while others used intermittent pressure. In addition, the number of months/cycles studied also differed.

In accordance with the current study a Turkish study^20^ also showed significant pain relief by acupressure at acupoint SP6, their sample size was smaller (n=34) and acupressure was applied only once a day. Similar to the present study was the age group and their study also comprised three MCs, p<0.01. The technique they used was different, applying intermittent pressure for six seconds and then a two-second interval, for a total of 10 minutes. They compared their acupressure group with a control group that applied pressure at a false point. A study in Egypt^21^ also showed a significant decrease in the VAS score after acupressure at SP6, (p<0.001).

The mean age was similar, while the intermittent acupressure technique was applied twice only on the first day, and in only one MC. In another study by Othman et al.^15^, the sample size was similar (n=50) but the age was less (15-20 years). The same acupoint SP6 was used for acupressure twice a day during the first three days of the MC in two months. They also used the VAS for pain measurement and found a significant reduction in pain, p<0.001. The constant acupressure technique was similar but the duration of pressure application was 10 minutes as compared to our two to five minutes. This highlights the fact that pain reduction is not statistically dependent on the duration of the pressure applied as similar results were obtained. They compared their intervention group with a control group who applied only a slight touch at SP6 instead of applying pressure.

A study conducted in Indonesia^22^, also showed similar results (p<0.001), where resembling the current study, a pre and post-acupressure study design was used but the sample size was less, n=15 junior high school students. They compared acupressure at SP6 for PD with abdominal stretching exercises. The students applied acupressure for two minutes but only once on the first day of one cycle only. In place of the VAS measurement, they used the Numeric Rating Scale (NRS) to document pain intensity. The mean difference of pre-acupressure and post-acupressure NRS was statistically significant (p<0.001).

Our findings are similar to the findings of Rahmi et al.^13^ despite the variation; n=16, age group 17-20 years, younger than the present study, NRS used for pain scoring and compared with a control group without intervention. Equally significant pain relief was found by Anggraini et al.^23^ who used three acupoints SP6, LI4, and ST36 while using the pretest-posttest approach with n=53 and age 16-18 years old. It is interesting to note in the various studies and various ethnicities, that irrespective of the duration, frequency, and type of acupressure, statistically significant pain reduction was attained in different age groups. In opposition to the present study, to the best of our knowledge, no other studies were found. However, one study on acupressure in postpartum perineal pain but *not on PD* was found. The study reported no relief in pain, and the *acupressure point was not SP6*, they used auricular acupressure.^24^ Hence we reject the null hypothesis.

This study did not show any significant correlation between the age of participants, BMI, age at menarche, and duration of MC with PD. It is in agreement with a study by Latif et al. regarding BMI,^5^ while a study by Khalid et al.^25^ found a significant correlation between age and BMI contrary to our findings.

The strengths of this study are; the design is a pre and post-intervention study that makes the findings more accurate as the pain threshold and baseline characteristics remain the same, and to the best of our knowledge acupressure at SP6 as an alternative therapy for PD has not been studied previously in Pakistan.

This study adds new information regarding the effectiveness of acupressure in dysmenorrhea in our population as ethnicity has been shown to affect pain perception. Similar studies in other countries have used different types and different durations of acupressure, this study has observed that irrespective of type and time duration of acupressure it is equally effective for pain relief in PD.

The importance and clinical relevance lie in the fact that acupressure provides a safe, effective, and low-cost method of pain relief for a recurring unavoidable ailment. Further research is required that will include women from all walks of life, all socio-economic statuses, and all educational levels including the rural areas.

### Limitations:

All participants were medical students hence the results cannot be generalized for the whole population.

## CONCLUSION

For women who wish to abstain from pharmacological products during menstruation due to various myths and beliefs, this study has provided an effective therapy for pain in PD. Acupressure is a method that is safe, easy, free from side effects, and cost-effective.

### Authors Contribution:

**SA:** conceived the idea, planned the study design, acquired and interpreted data, prepared the manuscript, statistical analysis and is responsible for the integrity of the research.

**QA:** Study design, helped in the interpretation of the results, and critically reviewed the article for intellectual content. Gave final approval for its integrity.

**RN: S**tudy design, data analysis, drafting the article, and revising it critically.

**MS:** Data collection and data analysis, writing, and Critical analysis.

## References

[1] Thakur P, Pathania AR (2022). Relief of dysmenorrhea–A review of different types of pharmacological and non-pharmacological treatments. Mater Today Proc.

[2] Sima RM, Sulea M, Radosa JC, Findeklee S, Hamoud BH, Popescu M (2022). The prevalence, management and impact of dysmenorrhea on medical students'lives—A multicenter study. Healthcare.

[3] Al-Matouq S, Al-Mutairi H, Al-Mutairi O, Abdulaziz F, Al-Basri D, Al-Enzi M (2019). Dysmenorrhea among high-school students and its associated factors in Kuwait. BMC Pediatr.

[4] Okuyan E, Gunakan E, Atac H, Çakmak Y (2021). The effect of turmeric on primary dysmenorrhea:Prospective case-control study. J Surg Med.

[5] Latif S, Naz S, Ashraf S, Jafri SA (2022). Junk food consumption in relation to menstrual abnormalities among adolescent girls:A comparative cross sectional study. Pak J Med Sci.

[6] Guimaraes I, Povoa AM (2020). Primary dysmenorrhea:assessment and treatment. Rev Bras Ginecol Obstet.

[7] Ashraf T, Riaz S, Atta S, Ikram A, Shehzadi HK (2020). Prevalence of dysmenorrhea and impact on young medical students;a cross sectional study on students of medical colleges of Lahore, Pakistan. Rawal Med J.

[8] Abdel-Salam DM, Alnuman RW, Alrwuaili RM, Alrwuaili GA, Alrwuaili EM (2018). Epidemiological aspects of dysmenorrhea among female students at Jouf University, Saudi Arabia. Middle East Fertil Soc J.

[9] Abreu-Sanchez A, Ruiz-Castillo J, Onieva-Zafra MD, Parra-Fernández ML, Fernández-Martínez E (2020). Interference and impact of dysmenorrhea on the life of Spanish nursing students. Int J Environ Res Public Health.

[10] Soderman L, Edlund M, Marions L (2019). Prevalence and impact of dysmenorrhea in Swedish adolescents. Acta Obstet Gynecol Scand.

[11] Murphy SL, Harris RE, Keshavarzi NR, Zick SM (2019). Self-administered acupressure for chronic low back pain:a randomized controlled pilot trial. Pain Med.

[12] Li T, Li X, Huang F, Tian Q, Fan ZY, Wu S (2021). Clinical efficacy and safety of acupressure on low back pain:A systematic review and meta-analysis. Evid Based Complement Alternat Med.

[13] Rahmi C, Rasima A, Irwan S, Amersha F (2022). The Effect of Acupressure Techniques on Menstrual Pain (Dysmenorrhea) in Nursing Students of Poltekkes Kemenkes Aceh. EAS J Nurs Midwifery.

[14] Chiarotto A, Maxwell LJ, Ostelo RW, Boers M, Tugwell P, Terwee CB (2019). Measurement properties of visual analogue scale, numeric rating scale, and pain severity subscale of the brief pain inventory in patients with low back pain:a systematic review. J Pain.

[15] Othman S, Aly S, Mady M (2019). Effect of acupressure on dysmenorrhea among adolescents. J Med Sci Res.

[16] Wong CL, Lai KY, Tse HM (2010). Effects of SP6 acupressure on pain and menstrual distress in young women with dysmenorrhea. Complement Ther Clin Pract.

[17] Tabari NS, Kheirkhah M, Mojab F, Salehi M (2020). An investigation of the effect of curcumin (turmeric) capsule on the severity and duration of dysmenorrhea in students of Iran University of Medical Sciences. J Evol Med Dent Sci.

[18] Elverisli GB, Armagan N, Atilgan E (2023). Comparison of the efficacy of pharmacological and nonpharmacological treatments in women with primary dysmenorrhea:randomized controlled parallel-group study. Ginekol Polska.

[19] Cherian MR (2020). Acupressure on Dysmenorrhea Pain Among Adolescent Girls. J Appl Sci.

[20] Dincer Y, Oskay U (2023). The Effect of Acupressure Applied to Sanyinjiao (SP6) on Primary Dysmenorrhea. Altern Ther Health Med.

[21] Awad NS, Mourad MH, Lamadah SM (2022). Effect of Spleen 6-point Acupressure on Pain Intensity among Late Adolescents Nursing Students with Primary Dysmenorrhea. Alex Sci Nurs J.

[22] Rabia Zakaria IH (2021). Effectiveness of abdominal stretching exercise and sanyinjiao acupressure to relieve dysmenorrhea pain. J Southwest Jiaotong Univ.

[23] Anggraini Y, Ekawati IW (2020). Acupressure therapy as a pain reliever for dysmenorrhea. Enferm Clin.

[24] Kwan WS, Li WW (2014). Effect of ear acupressure on acute postpartum perineal pain:a randomised controlled study. J Clin Nurs.

[25] Khalid M, Jamali T, Ghani U, Shahid T, Ahmed T, Nasir T (2020). Severity and relation of primary dysmenorrhea and body mass index in undergraduate students of Karachi:A cross sectional survey. J Pak Med Assoc.

